# 3D Strain Imaging of a Heterostructured GaInP/InP Nanowire Using Bragg Coherent Diffraction X-ray Imaging: Implications for Optoelectronic Devices

**DOI:** 10.1021/acsanm.4c06406

**Published:** 2025-01-28

**Authors:** Huaiyu Chen, Megan O. Hill, Magnus T. Borgström, Jesper Wallentin

**Affiliations:** † Synchrotron Radiation Research and NanoLund, Department of Physics, Lund University, 22100 Lund, Sweden; ‡ MAX IV Laboratory, Lund University, 22100 Lund, Sweden; § Solid State Physics and NanoLund, Department of Physics, Lund University, 22100 Lund, Sweden

**Keywords:** Bragg CDI, heterostructure nanowire, strain, coherency limits, angular correction

## Abstract

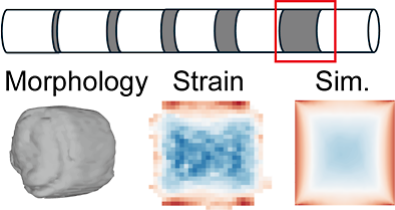

Imaging the strain in nanoscale heterostructures is challenging since it requires a combination of high strain sensitivity and spatial resolution. Here, we show that three-dimensional (3D) Bragg coherent diffraction imaging (BCDI) can be used to image the strain in a single InP segment within an axially heterostructured GaInP–InP nanowire. We use a 350 nm-diameter X-ray beam, which is smaller than the nanowire but larger than the 180 nm long InP segment. The intense nanofocused beam induced angular distortions, but these are successfully removed by a correction algorithm. Additionally, we show that data from multiple scans can be merged despite scan-to-scan variations. The reconstruction of the merged data set has a spatial resolution of approximately 14 nm, revealing the 3D morphology of the InP segment and its internal strain distribution. The measured strain shows qualitative agreement with finite element method simulations, but with slightly larger magnitude, which indicates a higher Ga composition than the nominal value. The 3D strain map suggests that the nanowire can accommodate the theoretically predicted lattice mismatch without exceeding the coherency limit. Continued development of robust BCDI measurements and reconstructions enables future studies of strain fields and coherency limits in axial nanowire heterostructures, which are critical for designing next-generation optoelectronic devices.

## 1 Introduction

In recent years, heterostructured semiconductor nanowires (NWs) have gained significant attention due to their excellent electronic and optoelectronic properties.^1−3^ Compared to bulk materials, NWs allow for interfacing of higher lattice mismatched material and more flexible heterostructure design. This is due to the high strain tolerance and three-dimensional (3D) nature of NW heterostructures.^4−6^ Consequently, axially heterostructured NWs are commonly used in lasers^7^ and solar cells^8,9^ because the energy bandgap can be controlled along the natural carrier transport direction.

The lattice mismatch at the interfaces between heterostructured junctions can lead to strain induced shifts of the band gap, which in some cases can be beneficial for device performance. However, if the interfacial strain is too large, it can result in defect formation and device degradation. Therefore, it is crucial to determine the strain limits of NW heterostructures, i.e., the coherency limits. Since simulations of heterostructured NWs are limited to simple NW geometries,^6^ the prediction of coherency limits is not as accurate as calculations for traditional thin films. As such, an additional experimental investigation of the strain state within more complex NW geometries is warranted.

Transmission electron microscopy (TEM) is a useful tool for characterizing NW heterostructures, but it is typically constrained to two-dimensional (2D) projections. Furthermore, its strain resolution is limited, especially for thicker (>100 nm) NWs. X-ray diffraction (XRD), on the other hand, is highly sensitive to strain and has a penetration depth that is compatible with thicker device geometries. With developments in X-ray optics^10,11^ highly coherent hard X-ray probes can now be regularly produced to be less than 100 nm spot sizes, making XRD imaging a useful tool for mapping the strain in NWs. Consequently, there are many reports utilizing scanning XRD^12−17^ and Bragg projection ptychography (BPP)^18−21^ to image strain. Scanning XRD provides a strain map by analyzing small angular deviations in the diffraction patterns. However, its spatial resolution is limited by the focused beam size, which can result in an averaging effect that fails to accurately reflect strain in regions with high strain variation.^22^ BPP, a coherent diffraction imaging (CDI) method, uses a combination of scanning XRD and phase retrieval methods to achieve sub-beam resolution. We have previously demonstrated that the strain measured by 2D BPP^22^ can produce higher real space and strain resolution than scanning XRD^23^ and that the results correlated well with simulation results using the finite element method (FEM). However, neither of these two methods can measure the 3D strain, which is a severe limitation for NW heterostructures.

An alternative method is 3D Bragg ptychography (3DBP), which offers sub-beam resolution and can reveal the 3D strain distribution of an extended sample.^24−26^ However, this method requires a large X-ray dose due to the long collection time, which can introduce beam damage in NWs. Further, 3D BP has strong requirements for positional alignment and oversampling in both real and reciprocal space. In contrast, 3D Bragg CDI (BCDI) is a common coherent method employed for nanoparticles with smaller size than the beam.^27−33^ This technique allows for the assessment of the 3D internal displacement field with high spatial resolution after phasing the oversampled 3D diffraction pattern along the direction of a selected Bragg peak. 3D BCDI is generally not applicable to extended crystals like NWs, as it requires the object to be smaller than the focused coherent beam and the measured crystals to be well separated. However, some examples demonstrate the application of BCDI to NWs. Davtyan et al.^34^ have reconstructed 2D cross section reconstructions of core–shell heterostructure NWs using combination of 3D BCDI at diffraction and 2D ptychography and Hill et al.^35^ employed the 3D BCDI on extended core–shell heterostructure NWs by leveraging the separation of crystal materials twinning in reciprocal space.

In this work, we demonstrate that BCDI can be used for 3D strain imaging of single segments within an axially heterostructured NW, a structure of significant interest for advanced optoelectronic devices. More precisely, we characterize a Ga_0.21_In_0.79_P (hereafter termed GaInP) axial NW with epitaxial InP segments of various lengths inserted throughout the wire. The nominal mismatch of 1.52% is near the predicted limit for defect formation.^5^ The measurements were performed on a single, embedded InP segment, and FEM simulations of the NW strain were conducted as a reference. BCDI is made possible by using a beam which is larger than the InP segment but smaller than the NW length. The measurements show clear signs of angular movement during and between acquisitions of diffraction data, which is a common problem in BCDI and other nanoprobe XRD methods.^33,36,37^ To overcome this problem, we use a recently presented correction method based on the expand-maximize-compress algorithm^38−42^ to correct the movements. As a result, we are able to reconstruct in 3D the strain profile within the InP segment, showing good quantitative agreement with simulations.

## 2 Material and Methods

### 2.1 Theory

**Figure 1:**
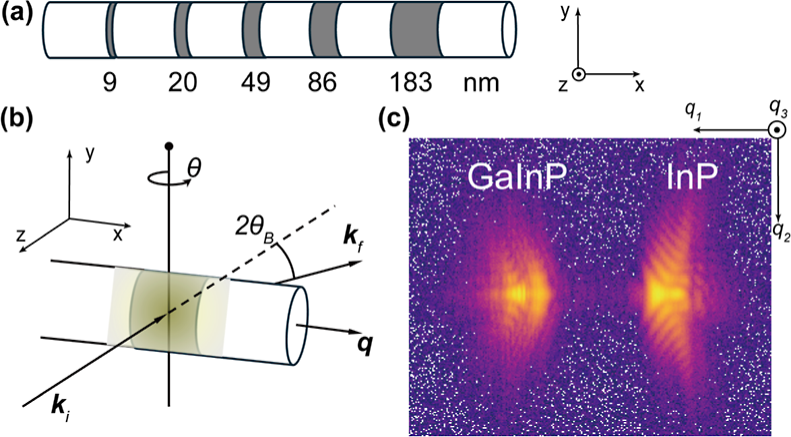
BCDI of an axially heterostructured NW. (a) Sketch of a single GaInP- InP NW. The NW has a barcode structure with 5 InP segments (gray) of different lengths. The axial direction of the NW is along *x* direction. (b) Experimental setup. A coherent beam (yellow highlight) is scattered by the largest InP segment and the neighboring GaInP segments with incident wave vector ***k***_*i*_ and exit wave vector ***k***_*f*_. The scattering vector ***q*** is along the axial direction of the NW. (c) Integrated diffraction pattern for one scan, with the GaInP Bragg peak on the left and the InP Bragg peak on the right. The detector (*q*_1_ × *q*_2_) is perpendicular to the exit vector ***k***_*f*_. The *q*_3_ direction corresponds to the rocking curve direction θ in real space.

In a BCDI experiment, as illustrated in Figure 1b, the Laue condition is satisfied when the scattering vector ***q*** = ***k***_*f*_ – ***k***_*i*_ coincides with one of the reciprocal lattice points ***G***_*hkl*_, where ***k***_*i*_ and ***k***_*f*_ represent the incident and exit wave vectors, respectively. The complex electron density ρ(***r***) of the measured object is
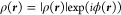
1where |ρ(***r***)| is the electron density, ***r*** is a real space vector, and ϕ(***r***) is the phase. The displacement field ***u***(***r***) describes local deviations from the ideal crystal lattice. The phase term ϕ(***r***) can then be defined as ϕ(***r***) = ***G***_*hkl*_·***u***(***r***),^43^ i.e., the projection of ***u***(***r***) onto the selected ***G***_*hkl*_. This displacement field modulates the complex electron density, and the resulting far-field diffraction pattern can be approximated as the Fourier transform of ρ(***r***). Consequently, the 3D diffraction intensity is represented as . Therefore, the far-field diffraction pattern obtained in the BCDI experiment encodes displacement field ***u***(***r***) projected along ***G***_*hkl*_, providing detailed insights into the internal structure of the object. However, since the phase information is lost, phase retrieval is necessary to reconstruct the displacement field. Strain can be described as the derivative of the displacement. Since the phase term corresponds to the displacement field along ⟨111⟩ *B* direction as mentioned above, the strain can be calculated as
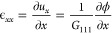
2

### 2.2 Material

As schematized in Figure 1a, the Ga_*x*_In_1–*x*_P – InP NWs studied in this work have five pure InP segments of varying lengths. These NWs were synthesized using the Au particle assisted growth mode via metal–organic vapor phase epitaxy^44^ and were grown in the ⟨111⟩ *B* direction of the zinc blende crystal structure. Each NW has an average diameter of 190 nm and a total length of approximately 2.2 μm. Previous work has indicated that the Ga content in these NWs is around *x* = 21%,^23^ with some variations. Prior to the BCDI measurement, these NWs were transferred to a Si_3_N_4_ membrane. The experimental setup was aligned to the ⟨111⟩ reflection of the NW, as this corresponds to the growth axis and facilitates straightforward alignment. During the measurement, the NWs, along with the substrate, were mounted on the rotation stage oriented horizontally.

### 2.3 Experiment

The BCDI measurements were performed at the NanoMAX beamline of the MAX IV Laboratory in Lund, Sweden. To fully cover the whole measured InP segment without illuminating the adjacent InP segments, the incoming X-ray beam, at an energy of 10 keV, was focused down to 350 × 350 nm with full coherent flux by reducing the entrance aperture to the Kirkpatrick-Baez mirrors.^45^ The In-L_3_ and Au-M_5_ X-ray fluorescence peaks collected by using a silicon drift diode detector were used to locate the NWs. Prior to measurement of the NWs, the beam was attenuated by 50% to avoid beam damage, resulting in a flux of 6 × 10^9^ photons/s. An Eiger 500k detector, mounted on a Kuka robot arm, was positioned at the Bragg condition for ⟨111⟩ reflection at 2θ_B_ = 21.09° at a distance of 1 m. A flight tube filled with He was used to reduce the air scattering. Figure 1a,b illustrates the NW heterostructure and diffraction geometry. The 3D diffraction volume was sampled by rotating the sample around rocking curve direction θ. A total of 180 frames were captured by the detector using step mode within a relative angular range [−0.9°, 0.9°] around Bragg angle θ_B_ to satisfy the Nyquist sampling condition. The exposure time per frame was 0.05 s. The detector frame was cropped to 200 × 200 pixels to isolate the InP diffraction pattern from the GaInP diffraction.

### 2.4 Angular Correction Method

In this study, an angular correction method^38,39^ was employed to mitigate angular distortions in the rocking direction, thereby improving data quality for the subsequent phase retrieval. The method generates a 3D diffraction volume that is iteratively updated by maximizing the likelihood, which is calculated using the probability mass function of a Poisson distribution between the input diffraction data set and the generated volume. To define the field of view, a real-space constraint is applied to the Patterson function of the generated volume. As a result, the algorithm returns both the corrected data set and a normalized probability matrix, which can be considered as the orientation trajectory. The code for the algorithm can be found in ref (39).

### 2.5 Simulation

To verify the reconstruction results from BCDI measurements, we performed an FEM simulation of a NW with a barcode structure consisting of GaInP and InP segments using COMSOL Multiphysics. The simulation employed a simple geometry, without bending, twisting, or composition gradient. The Ga composition was set to 21%, corresponding to a theoretical lattice mismatch of 1.52% between the GaInP and InP segments. This lattice mismatch was modeled as the initial strain state in the InP segments. The resulting displacement (phase) and the strain distribution of the NW, derived from the simulation, are presented in Figure S1 in the Supporting Information.

## 3 Results and Discussion

BCDI measurements were performed on the longest InP segment (180nm) within the GaInP–InP NW heterostructure. Figure 1c shows the diffraction patterns integrated along the rocking direction (which is *q*_3_ in the reciprocal space). The detector captured diffraction from both the targeted InP segment and the neighboring GaInP segments. Due to the difference in lattice parameter between two materials, their diffraction patterns are well-separated in reciprocal space. The intensity fluctuations with high frequency observed in the GaInP diffraction patterns result from the interference of the GaInP segments at both ends of targeted InP segment.

**Figure 2:**
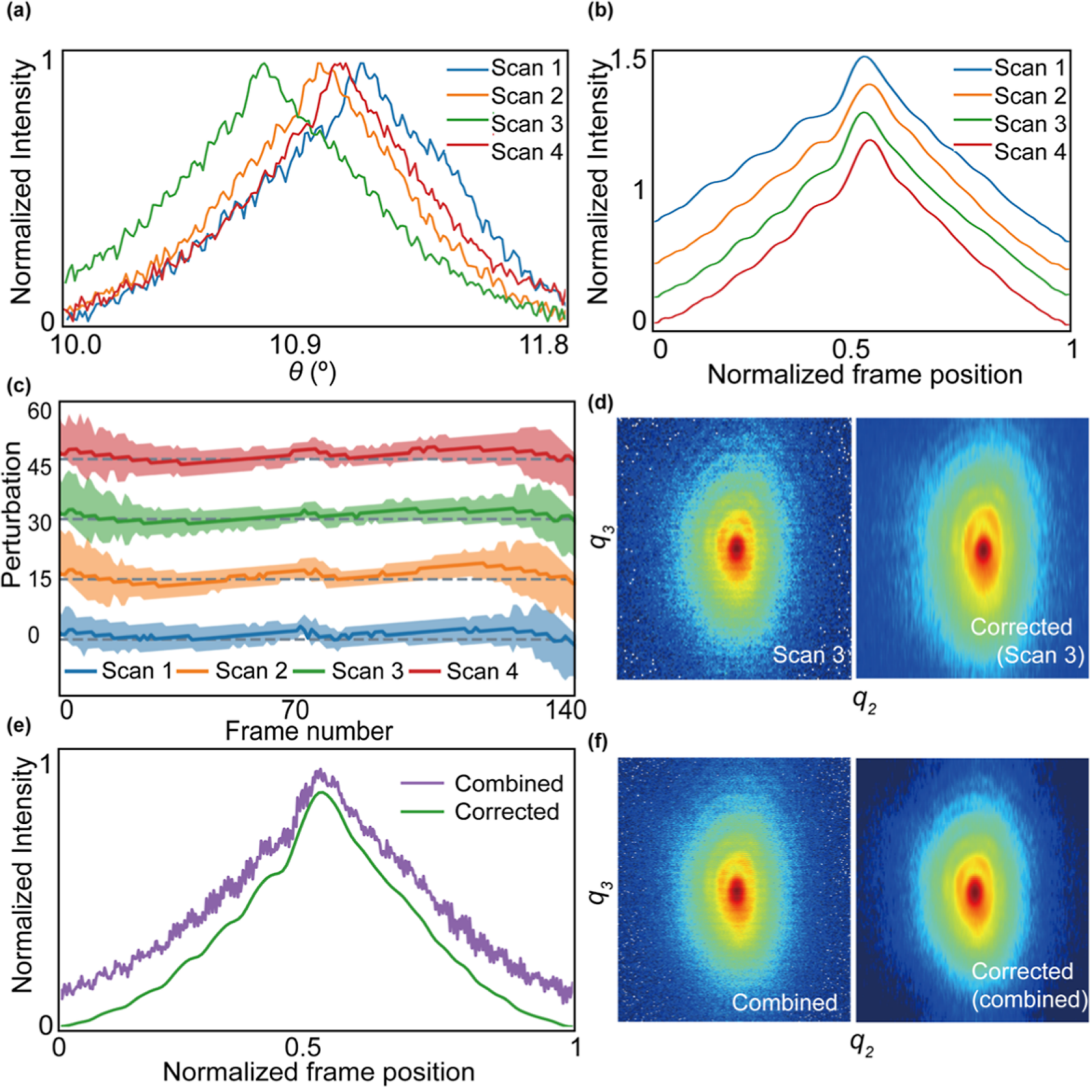
Correction of distorted measurements. (a) Rocking curves for 4 repeated scans, showing the normalized intensity as a function of rocking angle θ, on a logarithmic scale. (b) Rocking curves of the corrected data sets, processed using the correction algorithm. A vertical shift of 0.15 was applied for clarity. (c) Estimated perturbation distribution (colored shaded area) for each scan. The perturbation was calculated from the probability matrix with a threshold of 10^–3^. The colored solid line represents the peak position of the distribution for each frame. A shift of 15 was applied for visualization. (d) Integrated diffraction pattern along *q*_2_ direction for the example of Scan 3 and its corresponding corrected data set. (e) Rocking curves for the combined data set and the corresponding corrected data set. (f) Integrated diffraction pattern along *q*_2_ direction for the combined data set and its corrected data set.

To investigate the stability of the acquisitions, multiple nominally identical BCDI measurements on the same InP segment were performed in a sequence. A filter was applied to limit the flux, as nonreversible changes were observed at the full flux. Figures 2a and S2 show the rocking curves for each individual measurement. Clear angular shifts in the rocking curve direction (θ) are observed. As the NW is only fixed to the Si_3_N_4_ window with weak van der Waal forces, the beam pressure exerted by the incident X-rays can result in a sufficient torque to rotate the NW, an effect that is commonly observed in nanoparticles under nanofocused X-ray illumination.^36^ The effect might not only cause shifts at the beginning of the measurement but also result in shot-to-shot angular movements. It is worth noting that the angular shifts became more pronounced when longer counting times were used, emphasizing the need for shorter exposure durations to mitigate these effects. On the other hand, the overhead per point in a step scan makes it challenging to use very short counting times.

Furthermore, periodic fluctuations with a period of 0.05° can also be observed in each rocking curve, corresponding to the “stripes” on the example integrated diffraction pattern shown in the left panel of Figure 2d. We believe these “stripes” result from an interference effect caused by diffraction between closely spaced InP segments in the NW with the GaInP segment positioned in between. This effect is likely due to excitation by the long tail of the focused X-ray beam. The spacing of 0.05° in the rocking direction corresponds to approximately 370 nm when considering the experimental geometry, which is similar to the length of a single GaInP segment. This alignment strongly suggests that the interference arises from the InP segments, separated by the GaInP segment.

Both the possible angular drifts and interference will have a significant impact on the subsequent reconstruction. An angular correction algorithm,^38,39^ however, has proven to be a robust and reliable method to mitigate the effect from angular distortion in rocking direction θ. Beyond correcting for angular error, this algorithm applies an envelope to the Patterson function of the diffraction pattern, which enables it to potentially mitigate the interference effect by selecting the main features in the Fourier domain of the diffraction pattern.

Prior to the correction by the algorithm, each measurement was centered based on the peak position and cropped to 141 frames. The same set of parameters (envelope factor as 0.25, β as 2 × 10^–3^) was applied to each measurement in the algorithm. We set a tight envelope to better address the interference effect. Subsequently, the algorithm generated the corrected diffraction volume of 128 frames for each measurement along with the final probability matrix. This probability matrix can be used to determine the orientation trajectory.

Figure 2b shows the rocking curves for the corrected data sets of each scan. The rocking curves exhibit similar characteristics, such as an asymmetric shape and consistent positions of the local maxima. The observed asymmetry in the corrected rocking curves arises directly from the asymmetric strain distribution within the NW, as indicated by the diffraction pattern in Figure 1c. The consistency of these features shows that the correction algorithm effectively mitigated the angular distortions of the experimental data. The perturbation distribution obtained from the probability matrix (orientation trajectory) is presented in Figure 2c. It shows each measured data set experienced subtle angular distortion, primarily around the Bragg position (the middle of the rocking curves). The truncated behavior and linear trend observed in each perturbation distribution are due to the tight envelope, as discussed in our previous work.^39^ The integrated diffraction pattern of the corrected data set, corresponding to the example data set, is shown in the right panel of Figure 2d. The algorithm effectively removes the periodic stripes while preserving the primary features of the diffraction pattern.

Given that all the measurements were performed on the same InP segment, and their corrected data set share similar characteristics despite clear angular shifts, it is natural to consider merging the measurements and applying the algorithm on the combined data set. In general, merging the data sets can enhance the signal-to-noise ratio and provide a rich information redundancy for the algorithm, which could improve the data quality. Therefore, scans 2–4 were selected to be merged into a combined data set before correction, after coarse alignment using their peak positions. Scan 1 was excluded due to its substantially broader fwhm in the rocking curve. Figure 2e shows the rocking curve for the combined data set, while the upper panel in Figure 2f illustrates the integrated diffraction pattern. As with each individual measurement, high-frequency interference can be observed. After the correction by the algorithm, using a support factor of 0.12 and β of 3 × 10^–3^, a corrected data set with 128 frames was generated. The main features of its rocking curve align well with the combined data set but without interference, similar to the corrected data set of each individual measurement. We also attempted to combine data sets after correction, but this did not work well. Although the corrected data sets exhibited similar features, minor differences induced artifacts during direct combination.

A phase retrieval algorithm is necessary to numerically reconstruct the phase information.^46^ Here, we employed the PyNx software^47^ to perform the phase retrieval on our corrected data sets. Prior to that, the corrected data sets were cropped by 10 frames at both ends to eliminate possible effects from the tight envelope. The same reconstruction process of PyNx, consisting of 600 relaxed averaged alternating reflection^48^ iterations followed by 200 error-reduction^49,50^ cycles and shrink-wrap^51^ support threshold with amplitude coefficient 0.2–0.35, was applied on each corrected data set. The initial support was derived from the autocorrelation of the input corrected data set. From 1000 reconstructions, the 20 with the highest free log-likelihood values were selected and averaged to produce the final reconstruction.

**Figure 3:**
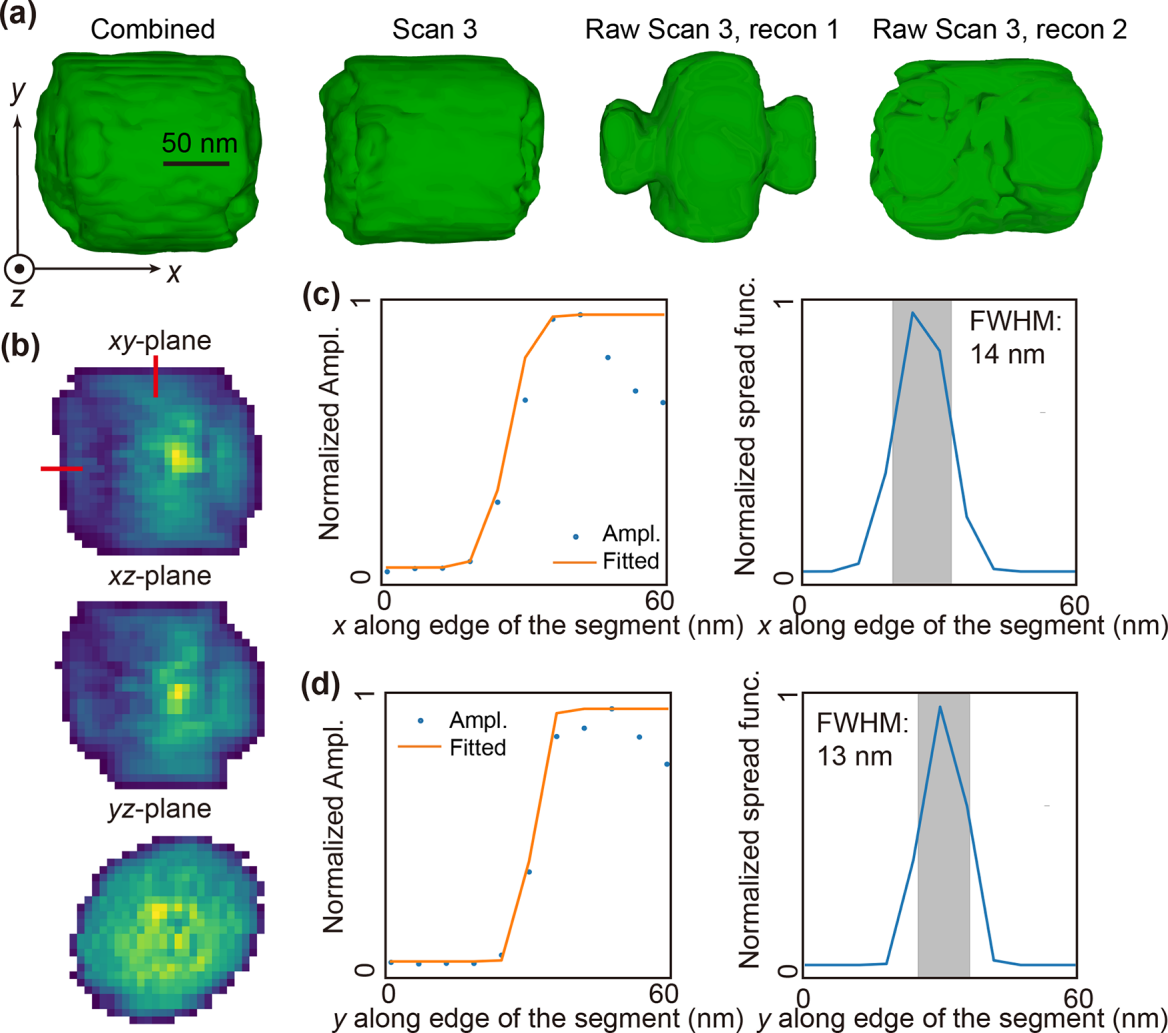
Phase retrieval results. (a) Reconstructed morphologies for corrected (left figures) and uncorrected/raw (right figures) data sets. The morphologies are visualized using amplitude isosurfaces with a threshold set at 0.1 of the maximum value for each reconstruction. The support threshold for the reconstruction of scan 3 raw data with autocorrection (recon 1) initial support was 0.25–1.0. Another reconstruction of scan 3 raw data (recon 2) with fixed cylindrical support. (b) Cross-sectional views for reconstructed amplitude distribution of the combined data set are shown for the *xy*-plane, the *xz*-plane, and the *yz*-plane. The normalized amplitude maps are color-coded from 0 to 1. (c) Resolution estimation of reconstruction in *x* direction. (d) Resolution estimation of reconstruction in *y* direction. The line outs (dots) from the amplitude edge in both directions, highlighted by red lines in the *xy*-plane in (b), fitted with error functions (orange solid line). The full-width half maximums (fwhm, gray region) of the derivatives of the error functions demonstrate the resolutions in both directions.

The reconstructed morphologies after geometry rectification^52^ are shown in Figure 3a. The voxel size for the reconstructions is 6.6 nm. The estimated spatial resolutions for each reconstruction are about 14 and 13 nm in the *x*- and *y*-directions, respectively (Figure 3c,d). For comparison, we also performed phase retrieval on the uncorrected (raw) data from an example measurement (scan 3) using both a fixed cylinder support and autocorrelation support (details in Figure S4). Additionally, *xy*-, *xz*-, and *yz*-plane cuts of the reconstructed amplitudes for the corrected data sets are presented in Figure S3.

Compared with the reconstruction from the uncorrected data, the reconstructions shown in Figure 3a accurately reproduce the 3D shape of the input, retaining the cylindrical features. The improvement in reconstruction quality is significant after correction. The Fourier shell correction,^53,54^ detailed shown in Figure S5, indicates the similarity between the reconstruction of corrected combined data set and the reconstructions from the other corrected data sets. The reconstructed morphologies exhibit similar features: Instead of flat interfaces, the middle section is slightly longer than the region near the surface. The reconstructed amplitudes displayed in Figures 3b and S3 show two “arc”- shaped local minima in the *xy*- and *xz*-planes, as also observed using measured and simulated BPP in our previous work.^22^

All of the reconstructions show a tilt or skewing in the *yz*-plane, which does not agree with TEM images of the NWs and suggests potential systematic errors. As discussed above, the angular distortions could be caused by the photon pressure of the X-ray beam.^36^ We, therefore, simulated a systematic angular shift that is proportional to the diffracted intensity. Note that the scattering vector in our case, unlike ref (37), is parallel with the NW axis, thus requiring an indirect mechanical coupling to the local bending and tilting around the rocking curve direction. These simulations, detailed in Figure S6, indicate that such systematic errors could indeed lead to the observed skew. The simulation is not intended to be a quantitative comparison but rather qualitatively shows how photon pressure can lead to the observed skewing. Although the correction algorithm significantly improved the reconstructions, it does not fully account for these long-range effects since it fundamentally relies on correlations between frames.

**Figure 4:**
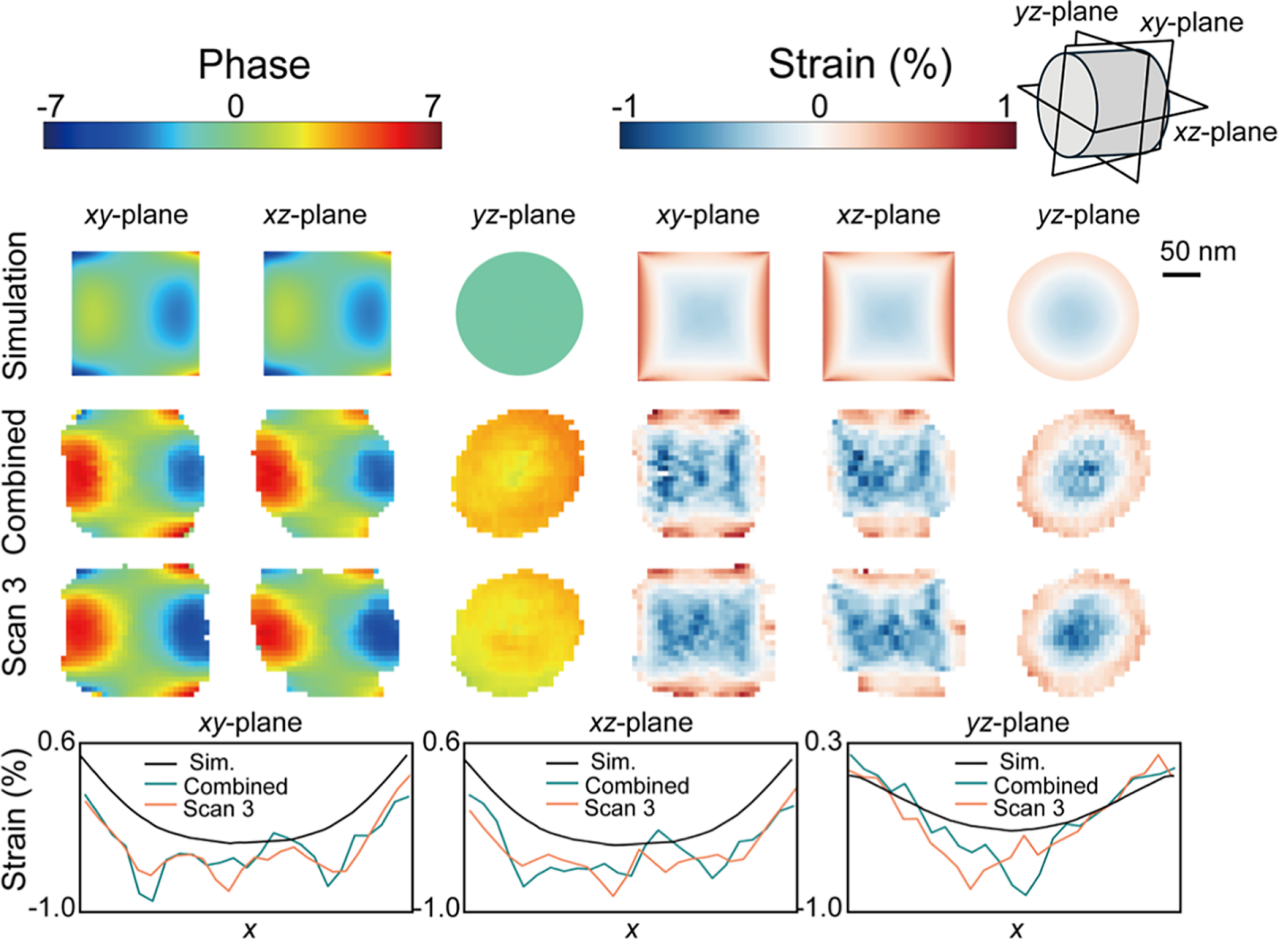
Phase (unwrapped) and strain maps of the largest InP segment from BCDI measurements and simulations. Each row represents different data set or simulation, and each column corresponds to a different cross-sectional plane. The combined data set was obtained by merging the 3 individual measurement data sets. Cross-sectional views for phase and strain distribution are shown for the central *xy*-plane, *xz*-plane, and *yz*-plane. The bottom panels display line outs of the strain distribution for each data set across the cross sections: in the center for the *xy*- and *yz*-planes and slightly offset from the center for the *xz*-plane.

We now examine the phase and strain distributions of the InP segment. Figures 4 and S7 display the phase and strain distributions of each reconstruction alongside simulated results, while the full FEM results are shown in Figure S1. The strain is derived from the phase according to eq 2. All of the reconstructed phases exhibit similar distributions in the *xy*- and *xz*-planes: a harmonic feature, varying from positive to negative, with peak values appearing at each corner. Surprisingly, the reconstructed phases in the middle area of *xy*- and *xz*-planes from both individual measurements and the combined data set show greater magnitude, ranging from about −3.8 to +5.6, compared with the simulation, which ranges from about −3.5 to 1.1.

The strain distributions calculated from the phase also show excellent agreement with the simulation, with a sign switch between the edges and the middle region. The strain distribution along the edge exhibits a clear gradient, with the highest strain at both ends gradually decreasing toward the center. The strain distributions in *xz*-plane is more asymmetric than in *xy*-plane. Along with the morphologies presented above, we believe this asymmetry is because of the slightly inhomogeneous Ga distribution that led to a slightly higher lattice mismatch on one side.^23,55^

The reconstructed strain distributions shown in Figures 4 and S7 appear slightly grainy in the middle region, likely due to fluctuations in the phase distributions. To minimize the impact of these local maxima, line outs of the strain distribution for both Scan 3 and the combined data set, as well as the simulation, are displayed in Figure 4. Overall, the reconstructed strains from both the combined data set and scan 3 are consistent, showing a higher strain state in the middle region than the simulation across all cross sections. In the *xy*- and *xz*-planes, the average strain in the middle area is similar, around −0.5%, while the average simulated strain is about −0.3%. In the *yz*-plane, the average reconstructed strain is about −0.4%, showing a smaller deviation from the simulated value of −0.25%. Thus, the measured strain is generally larger than the simulated strain since the phase variation is larger. We believe that this difference is due to the actual averaged Ga composition of the specific NWs being slightly higher than the averaged nominal averaged composition (21%),^23^ leading to a larger lattice mismatch.

## 4 Conclusions

We have demonstrated BCDI as a tool for reconstructing the complex 3D internal strain distribution within single segments embedded in an extended semiconductor NW heterostructure. We used an X-ray beam that is smaller than the NW but larger than the InP segment. In this study, the angular correction algorithm not only helped mitigate the angular drift observed in the rocking curve direction but also eliminated interference effects. This method significantly improved the data quality and subsequent phase retrieval reconstruction.

The reconstructed phase and strain distributions show a good agreement with FEM simulations, indicating that the NW can accommodate the theoretically predicted lattice mismatch^5^ without exceeding the coherency limit. The higher values observed in the phase and strain distributions imply a higher Ga composition than previously calculated in earlier studies.^22,23^ These findings demonstrate that BCDI can provide accurate 3D measurements of strain in NW heterostructures, offering valuable insights into strain engineering.

We merged three data sets into one and applied the angular correction algorithm to generate a new combined data set. The analysis of this combined data set showed results similar to the individual data sets and the simulation, indicating the effectiveness and robustness of this approach. We were able to combine only three scans, but, in principle, far more scans could be used. Each scan had a total exposure time of only 9 s, so it should be possible to acquire and combine hundreds of scans. We believe that this approach has significant potential for other scenarios, particularly those involving samples that are unstable in the beam but do not degrade over time. In such cases, each individual BCDI measurement might yield sparse useable diffraction patterns due to extremely short exposure times, but it would be feasible to repeat the BCDI measurements multiple times. By combining data from multiple measurements of the same object, one could enhance data quality and improve overall resolution. Using multiple frames with a short exposure time could also mitigate the problem of movement within a single frame.

In summary, our study demonstrates the significant potential of BCDI for strain imaging in heterostructured NWs, enabling the design and optimization of next-generation optoelectronic devices. By advancing the understanding of strain fields and coherency limits in NW heterostructures, BCDI provides a pathway for improving device performance in applications such as solar cells, lasers, and optoelectronic technologies.

## Supporting Information Available

Finite element method simulations of displacement and strain fields in GaInP/InP NWs; rocking curves of raw data sets; reconstructed amplitude maps of corrected data sets; reconstructed amplitude, phase, and strain maps of uncorrected data sets; Fourier shell correlation analysis of reconstructions; systematic error simulation from beam pressure; and reconstructed phase and strain of corrected data sets (PDF)
