# Supplementary material for: 3D Strain Imaging of a Heterostructured GaInP/InP Nanowire Using Bragg Coherent Diffraction X-ray Imaging: Implications for Optoelectronic Devices
Source: ACS Appl Nano Mater. 2025 Jan 28;8(5):2310–8. doi: 10.1021/acsanm.4c06406 (PMC11811927; doi:10.1021/acsanm.4c06406)
Supplement: Supplementary file 1 — an4c06406_si_001.pdf [file an4c06406_si_001.pdf]

# Supporting information

# 3D Strain Imaging of a Heterostructured GaInP/InP Nanowire Using Bragg Coherent Diffraction X-Ray Imaging: Implications for Optoelectronic Devices

Huaiyu Chen[1*], Megan O. Hill[2], Magnus T. Borgström[3], Jesper Wallentin[1]

[1]Synchrotron Radiation Research and NanoLund, Department of Physics, Lund University, 22100 Lund, Sweden

[2]MAX IV Laboratory, Lund University, 22100 Lund, Sweden

[3]Solid State Physics and NanoLund, Department of Physics, Lund University, 22100 Lund, Sweden

[*]Correspondence e-mail: huaiyu.chen@sljus.lu.se

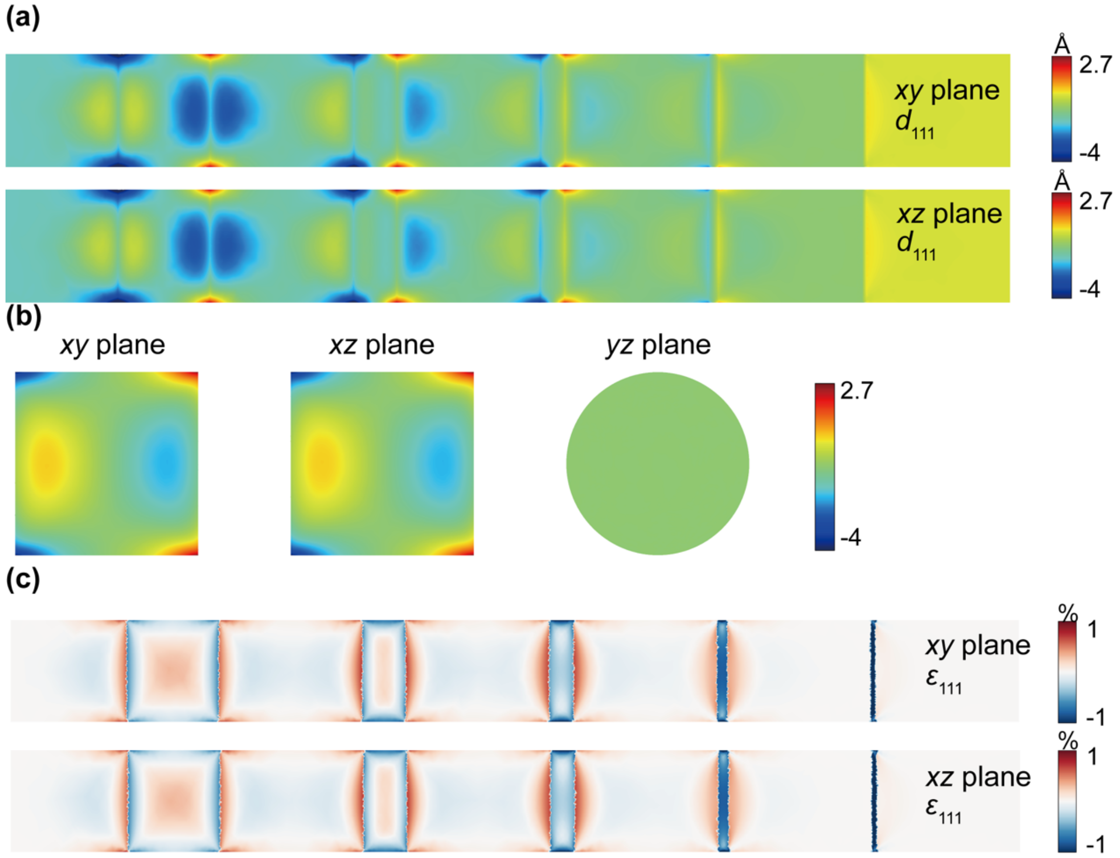


**Figure S1** Finite element method simulation of a multi-segment GaInP/InP nanowire heterostructure. (a) The overall displacement field $\boldsymbol{u}_{111}$ in the *xy*- and the *xz*- planes as calculated from FEM simulation. (b) The displacement field $\boldsymbol{u}_{111}$ of the measured largest InP segment in three cross sections. (c) The entire simulated strain distribution $\epsilon_{111}$ in the *xy*- and the *xz*- planes.

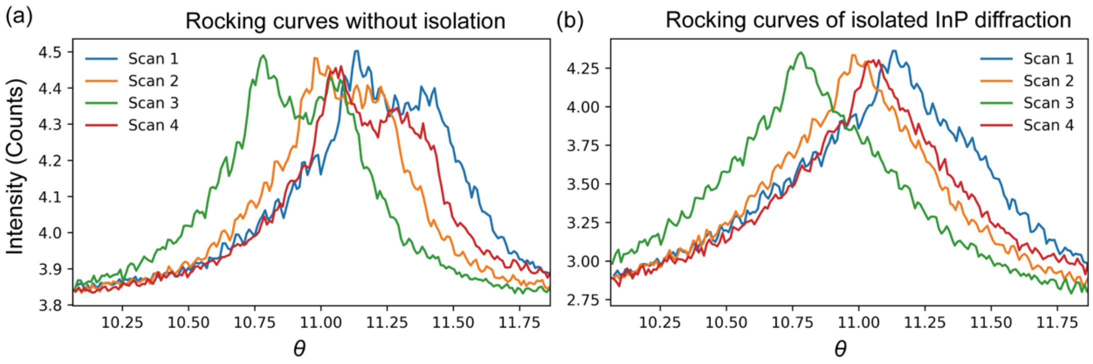


**Figure S2** Rocking curves for each individual scan without normalization. (a) Original rocking curves of both GaInP and InP diffractions. (b) Original rocking curves of the isolated InP diffraction pattern. The maximum intensity of each scan remains similar, indicating the lack of beam damage.

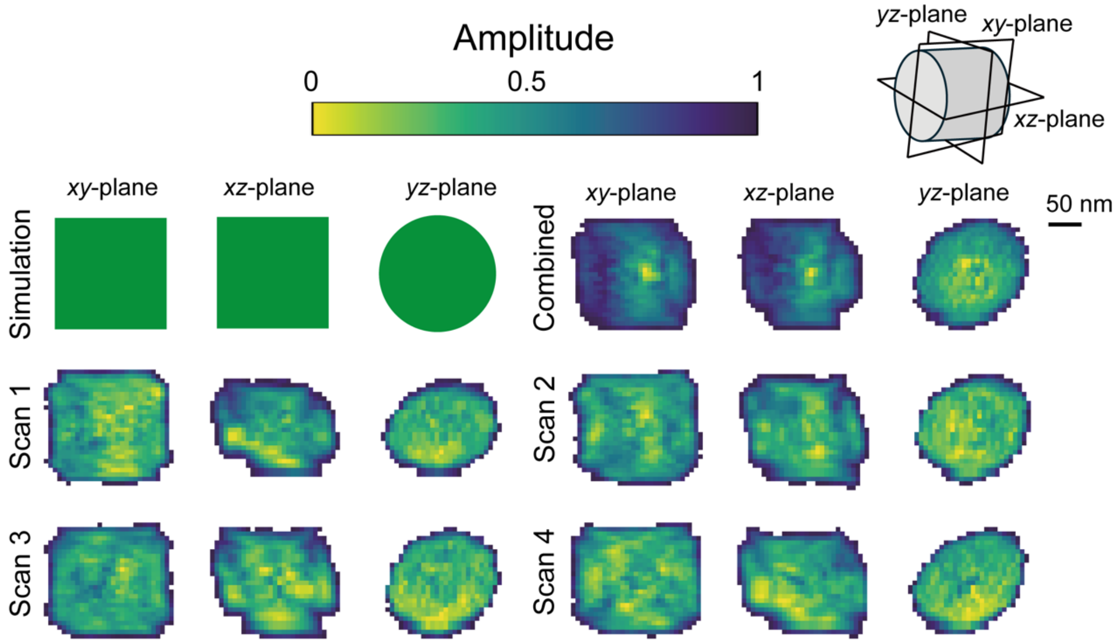


**Figure S3** Amplitude maps of the largest InP segment from BCDI measurements and Simulations. Cross-sectional views for amplitude distribution are shown for the $xy$-plane, the $xz$- plane and the $yz$- plane. The normalized amplitude maps are color-coded from 0 to 1.

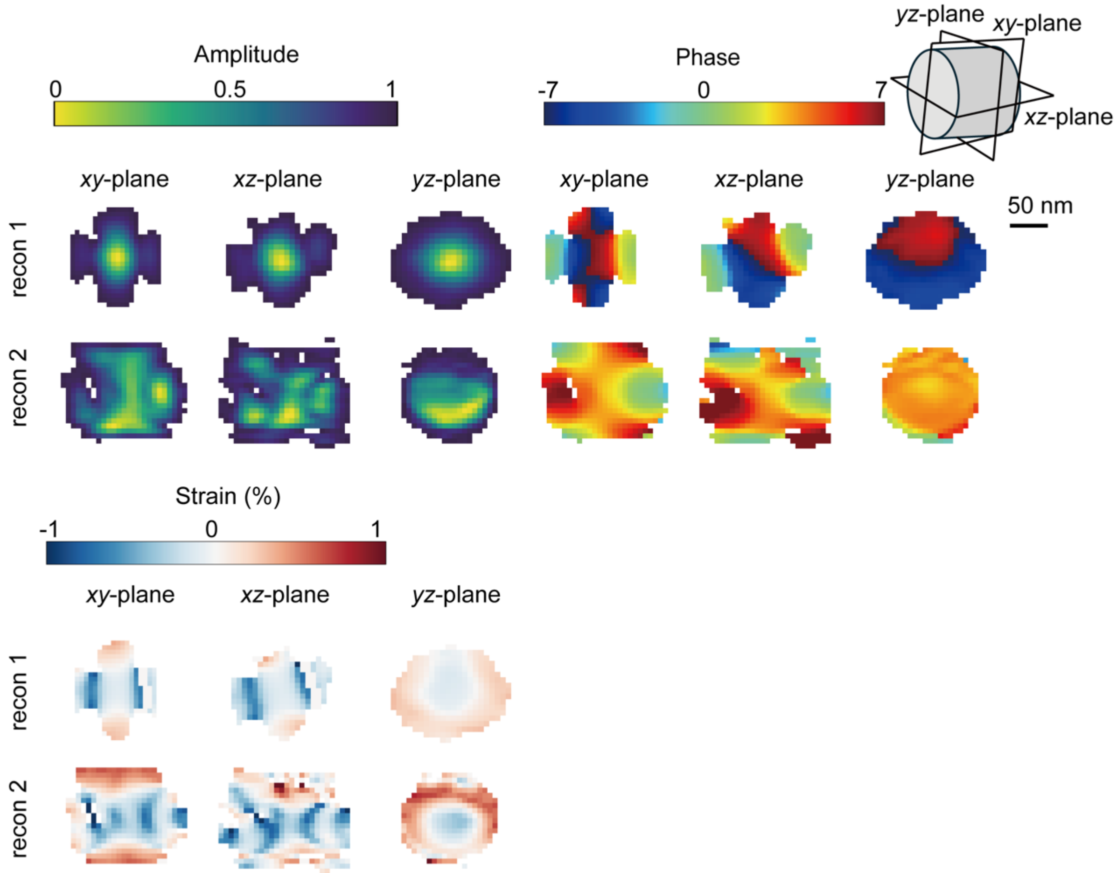


**Figure S4** Amplitude, phase (unwrapped), and strain maps from recon 1 and recon 2 (raw uncorrected dataset). Each row represents a different dataset, and each column corresponds to a different cross-sectional plane. Cross-sectional views of the phase and strain distributions are shown for the $xy$-plane, the $xz$-plane, and the $yz$-plane. Thresholds were applied to recon 1 and recon 2 to reduce noise: 10% for recon 1 and 5% for recon 2. The normalized amplitude maps are color-coded from 0 to 1, the phase maps are color-coded from -7 to 7, and the strain maps are color-coded with a range from -1% to 1%.

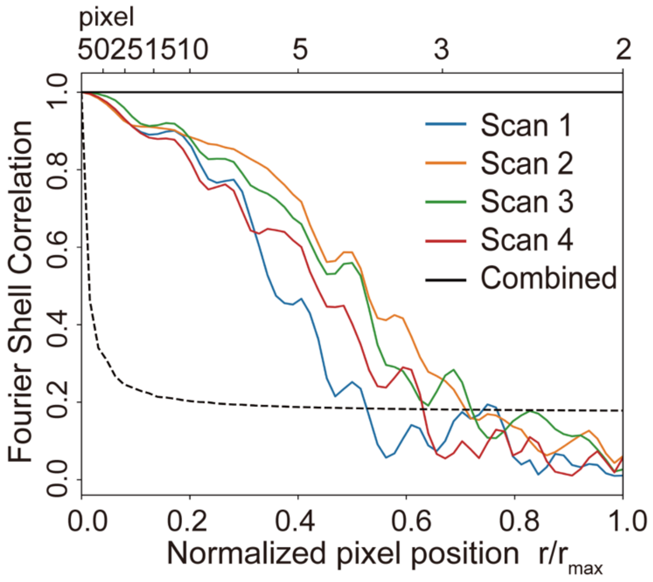


**Figure S5** Fourier shell correlation (FSC) for each reconstruction from individual scans relative to the reconstruction from the combined dataset. The black dashed line represents the 1/2-bit threshold, used to assess the similarity of each reconstruction. It is not surprising to see that Scans 2-4 have higher similarity than Scan 1 (around 4.1 pixels), as Scans 2-4 comprise the combined dataset.

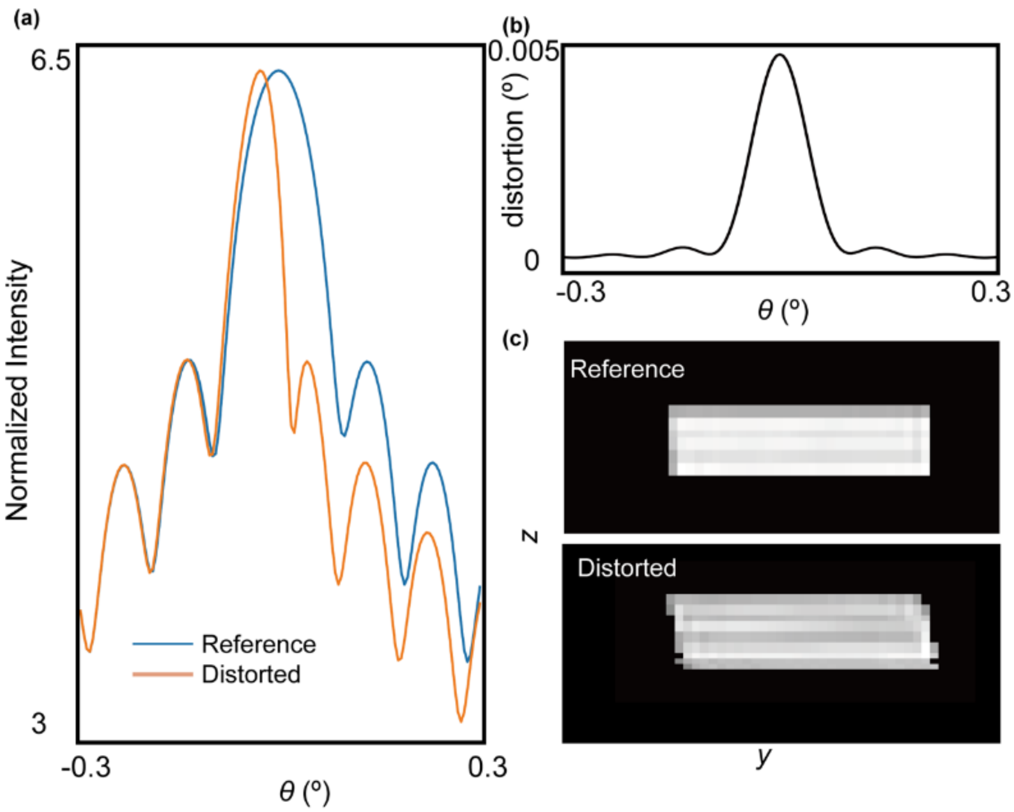


**Figure S6** Simulation of the systematic error from photon pressure. The simulation is not intended to be a quantitative comparison but rather shows how photon pressure qualitatively can lead to the observed skewing. To simplify the phase retrieval, we simulated a rectangular sample without strain. We added an angular deviation which was proportional to the scattering intensity. (a) Rocking curves for the reference dataset and the dataset with systematic error. (b) The angular deviation applied during the simulation. (c) Reconstructions after geometry rectification. The presence of systematic error leads to a noticeable skew in the corresponding rocking direction, which can be compared with the NW reconstructions in Figure 3 and 4.

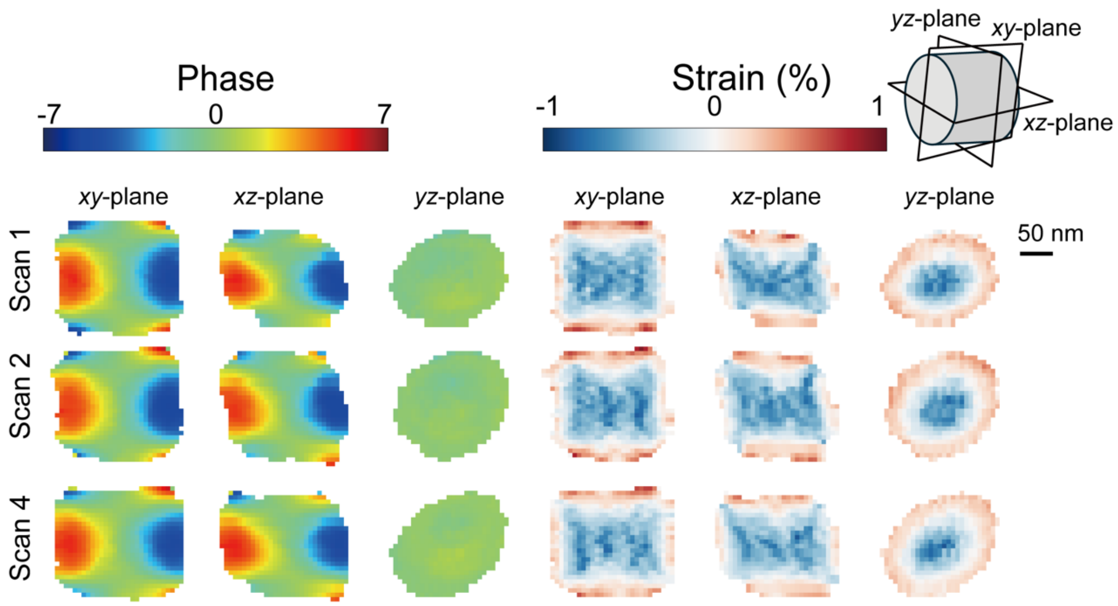


**Figure S7** Phase (unwrapped) and strain maps from scan 1, 2 & 4. Each row represents a different dataset, and each column corresponding to a different cross-sectional plane. Cross-sectional views for phase and strain distribution are shown for the $xy$- plane, the $xz$- plane and the $yz$- plane. The phase maps are color-coded from -7 to 7. The strain maps have a color scale varying from -1% to 1%.
